# Social and Experiential Drivers of Adolescent Alcohol Use: Evidence from an Exploratory Concept Mapping Study

**DOI:** 10.3390/children13030426

**Published:** 2026-03-20

**Authors:** Sheila Ares-Maneiro, Albert Espelt, Lucía Antelo-Iglesias, Ester Teixidó-Compañó, Marina Bosque-Prous, Èlia Colomeda-Cortada, Lucía Moure-Rodríguez, Ainara Díaz-Geada

**Affiliations:** 1 Department of Psychiatry, Radiology, Public Health, Nursing and Medicine, University of Santiago de Compostela, 15705 Santiago de Compostela, Spain; saresm@uoc.edu (S.A.-M.); lucia.antelo.iglesias@rai.usc.es (L.A.-I.); lucia.moure.rodriguez@usc.es (L.M.-R.); ainara.geada@usc.gal (A.D.-G.); 2 Epi4health Research Group, Faculty of Health Sciences, Universitat Oberta de Catalunya, 08018 Barcelona, Spain; elia.colomeda@uab.cat; 3 Epi4health Research Group, Facultat de Psicobiologia i de Metodologia en Ciències de la Salut, Universitat Autònoma de Barcelona (UAB), 08193 Barcelona, Spain; albert.espelt@uab.cat; 4 CIBER de Epidemiologia y Salud Pública, 28029 Madrid, Spain; 5 Department of Epidemiology and Methodology of Social and Health Sciences, Faculty of Health Sciences at Manresa, Universitat de Vic–Universitat Central de Catalunya (UVic-UCC), 08241 Manresa, Spain; eteixido@umanresa.cat; 6 Epi4health Research Group, Institute for Research and Innovation in Life and Health Sciences in Central Catalonia (IRIS-CC), Universitat de Vic–Universitat Central de Catalunya (UVic-UCC), 08500 Vic, Spain

**Keywords:** adolescence, alcohol consumption, consumption motivations

## Abstract

**Highlights:**

**What are the main findings?**

- Social factors, such as peer influence, social pressure, and the enjoyment-related motives, emerged as the most salient motivations for alcohol use during adolescence.
- Influences related to advertising, social media, and online content were perceived as less frequent and less important drivers of alcohol consumption.

**What are the implications of the main findings?**

- Reducing the social normalization of alcohol and promoting accessible alcohol-free leisure alternatives may strengthen prevention efforts.
- Enhancing adolescents’ social and emotional skills may increase the effectiveness of public health strategies aimed at reducing alcohol use.

**Abstract:**

**Background/Objectives**: To identify and prioritize adolescents’ motivations for alcohol consumption using a participatory qualitative approach. **Methods**: We conducted a concept mapping study with 39 adolescents aged 15–16 years from a public secondary school in Santiago de Compostela, Spain. Participants generated statements in response to a focal question about reasons for drinking, grouped them into categories, and then rated each statement according to perceived frequency and importance using a five-point Likert scale. **Results**: A total of 41 statements were generated and organized into eight clusters: peer approval, influence, enjoyment, experimentation, fun, disinhibition, social pressure, and coping. Motivations related to fun, peer dynamics, and disinhibition received the highest ratings for both frequency and importance. In contrast, motivations linked to advertising, social media, and influencers were rated lowest. A strong positive association was observed between perceived importance and reported frequency across statements. **Conclusions**: Adolescents identified enjoyment and peer dynamics as the primary motivations for alcohol use, emphasising the significance of social influences in adolescent drinking behaviours. Despite the necessity for cautious interpretation of findings due to the context-specific nature of the sample, the results suggest that prevention efforts may benefit from the promotion of alcohol-free social environments, the strengthening of social-emotional skills, and the involvement of adolescents in preventive initiatives.

## 1. Introduction

Adolescence is defined as a period of rapid development during which significant physical, cognitive and social changes occur over a relatively short time span [1,2]. This developmental stage is characterized by an increased propensity for risk-taking behaviors, closely associated with the heightened impulsivity typical of this period [3]. These changes influence a multitude of health-related behaviours, which in turn affect young people’s social, academic and health outcomes [2,4]. In consideration of this context, the consumption of alcohol by adolescents is a matter of public health concern for several reasons. It is the most widely used psychoactive substance in this age group [5]. Furthermore, the consumption of alcohol during this critical developmental period, when young people are particularly susceptible to external influences and establish behavioral patterns that often persist into adulthood, is associated with a broad range of adverse outcomes [6]. Evidence indicates that the drinking behaviors among adolescents are influenced by a combination of personal, social, and commercial factors, reflecting the diverse influences that affect their attitudes and patterns of alcohol use [7]. Although adolescence is characterized by increasing independence and a gradual separation from the family, the family environment continues to play a crucial role in shaping adolescents’ attitudes towards alcohol and their drinking behaviors [8]. Furthermore, the dynamics of the family environment, peer relationships, and the broader structural, cultural, and environmental factors that influence adolescents have been shown to have a substantial impact on the attitudes and patterns of alcohol consumption observed among this age group [9,10,11,12].

In many societies, alcohol use is widely normalized and socially accepted [13] and is often associated with leisure, celebration, social bonding, stress relief, and the expression of autonomy [14,15]. The influence of industry-driven marketing strategies serves to further reinforce these associations, thereby increasing adolescents’ exposure to pro-alcohol messages across a variety of contexts [14,15,16]. Social media platforms represent an additional influential environment in which alcohol-related content is frequently shared, normalized, and sometimes glamorized, thereby shaping adolescents’ perceptions, social norms, and intentions regarding drinking [17,18]. These influences may be particularly pronounced among adolescents living in socially disadvantaged contexts, where alcohol use is prevalent and easily accessible, or where parental attitudes toward drinking are more permissive [8,9]. The normalization of alcohol within family, social and community contexts may further reduce adolescents’ risk perception, thereby limiting the effectiveness of informal protective factors [19]. Collectively, these factors increase the likelihood of early initiation of risky alcohol-related behaviors, which have consequences for physical health, emotional regulation, and social development [20,21].

The extant literature on alcohol consumption in adolescents has been predominantly shaped by quantitative, survey-based research aimed at identifying risk factors [20]. In contrast, qualitative studies are comparatively scarce and, when conducted, have tended to focus on young adult populations instead of adolescents [14,20,22,23]. This imbalance has resulted in a limited understanding of adolescents’ own perspectives on the motivations underlying alcohol use. In this sense, the aim of our study was to identify and prioritise the motivations behind adolescents’ alcohol consumption using a concept mapping approach.

## 2. Materials and Methods

### 2.1. Sample

This study was conducted at a public secondary school located in the city centre of Santiago de Compostela (Spain), situated in an area of medium socioeconomic status, using a concept mapping method [24]. The study population comprised 4th-year compulsory secondary education (ESO) students (N = 86). The final sample consisted of 39 adolescents aged between 15 and 16 years who provided informed consent signed by a parent or legal guardian; 60.5% of the sample were female. One participant was excluded from the analysis due to inconsistent responses in the questionnaire.

### 2.2. Procedure, Instruments and Data Analysis

We conducted data collection and analysis in accordance with the six main steps of the Concept Mapping methodology [25], which are outlined below.

*Step 1: Preparation*. The following focal question was employed: ‘What are the reasons that lead you to drink alcohol? Or, if you don’t drink alcohol, what do you consider to be the reasons for doing so?’

*Step 2. Statement generation*. (Session 1 of the data collection.) The adolescents were introduced to the research team and informed about the study objectives and the importance of their participation. In accordance with the age of the participants, written consent from both the adolescents and their legal guardians was obtained prior to their involvement in this study. To obtain a comprehensive understanding of the motivations for consumption, a brainstorming session was conducted. We provided students with a sheet of paper presenting the focal question and invited them to individually write down all ideas that came to mind in response. Following a brief period of reflection (10 min approx), we conducted the brainstorming session, in which all the ideas were collated and aggregated on the blackboard to facilitate group discussion and achieve consensus with the adolescents. In addition, we administered a questionnaire to assess alcohol consumption patterns, including hazardous drinking, as measured by the AUDIT-C instrument.

*Step 3. Classification and evaluation of statements*. (Session 2 of the data collection). Following the brainstorming session, we created a statement bank from the responses generated in session 1. In a subsequent session, all statements were presented to the adolescents and they were invited to cluster the items into groups and rate each statement according to its perceived importance and frequency using a five-point Likert scale (1 = very low importance/frequency; 5 = very high importance/frequency).

*Step 4. Data analysis and statement representation on maps*. Data from previous sessions were analysed using the open-source concept mapping software R-CMAP (https://haim-bar.uconn.edu/software/r-cmap/, accessed on 1 February 2025), which employs hierarchical cluster analysis to maximise within-cluster similarity and between-cluster differentiation. The data were conceptualised through the generation of representations, including point maps, cluster maps, mean ranking maps, and go-zone maps [26]. We first conducted descriptive analyses of the sample characteristics using SPSS v21. Subsequently, we calculated mean statement scores and performed gender-stratified analyses based on participants’ groupings and ratings.

*Steps 5 and 6. Interpretation of results*. A thorough review and interpretation of the maps generated through the analysis was conducted, employing a comparative analysis of alternative cluster solutions. The configuration that demonstrated the optimal alignment with the data was selected, with consideration given to the participants’ grouping patterns. The labelling of clusters was conducted in accordance with their constituent statements and the names proposed by participants.

### 2.3. Ethical Considerations 

This study was conducted in accordance with the Declaration of Helsinki and received approval from the Ethics Committee of Universidade de Santiago de Compostela (25 November 2022). We also obtained institutional approval from the participating schools. Before data collection, written informed consent from parents or legal guardians and assent from the adolescents were obtained.

## 3. Results

### 3.1. Descriptive Data Analysis

The sample consisted of 60.5% girls and 39.5% boys with a mean age of 15.1 years. As shown in Table 1, most participants lived with their parents or with their parents and siblings (87% of girls and 80% of boys). Most adolescents self-perceived their health as either “very good” (43.5% of girls; 53.3% of boys) or “good” (43.5% of girls; 40% of boys). Statistically significant differences were not observed between girls and boys. Overall, 60.9% of girls and 71.4% of boys reported never having consumed alcoholic beverages. Furthermore, 65.2% of girls and 66.7% of boys indicated that they consumed no drinks during a typical drinking occasion, whereas 6.7% of boys reported consuming 10 or more drinks. In relation to binge drinking (defined as the consumption of six or more drinks on a single occasion), 73.9% of girls and 86.7% of boys reported never engaging in this behaviour.

### 3.2. Statements and Clusters

**Figure 1:**
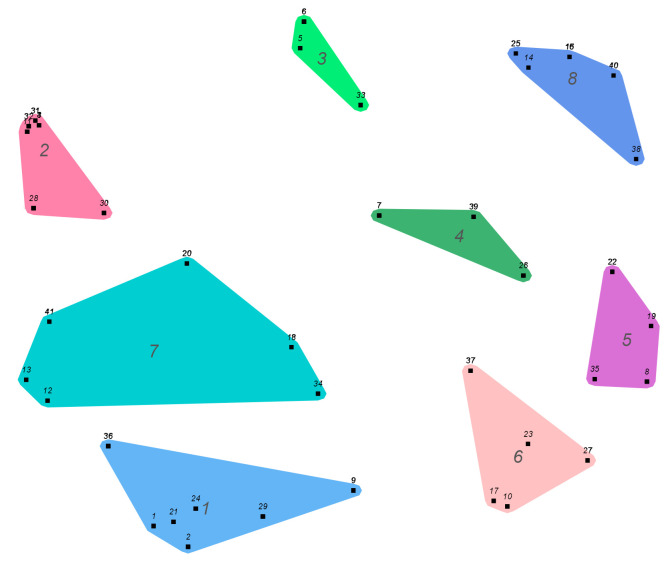
Cluster map. Cluster 1, peer approval; cluster 2, influence; cluster 3, enjoyment; cluster 4, experimentation; cluster 5, fun; cluster 6, disinhibition; cluster 7, social pressure; cluster 8, coping.

The brainstorming session conducted in Session 1 yielded 41 statements, which were grouped into 8 clusters (Figure 1): Cluster 1, peer approval; Cluster 2, influence; Cluster 3, enjoyment; Cluster 4, experimentation; Cluster 5, fun; Cluster 6, disinhibition; Cluster 7, social pressure; and Cluster 8, coping. The identification of several clusters reflected the conceptual proximity of specific statements. Within the same group, there were statements that were more closely related to each other. For example, Cluster 2 (“Influence”) captures two related dimensions: the impact of social media and public figures (statements 3, 4, 11, 31, and 32) and the influence exerted by the immediate social environment, particularly older siblings and friends (statements 28 and 30). Similarly, Cluster 7 (“Social pressure”) comprises two closely related dimensions: general social expectations (statements 12, 13, and 14) and peer pressure experienced in age-specific social contexts, such as parties (statements 18, 20, and 34).

**Figure 2:**
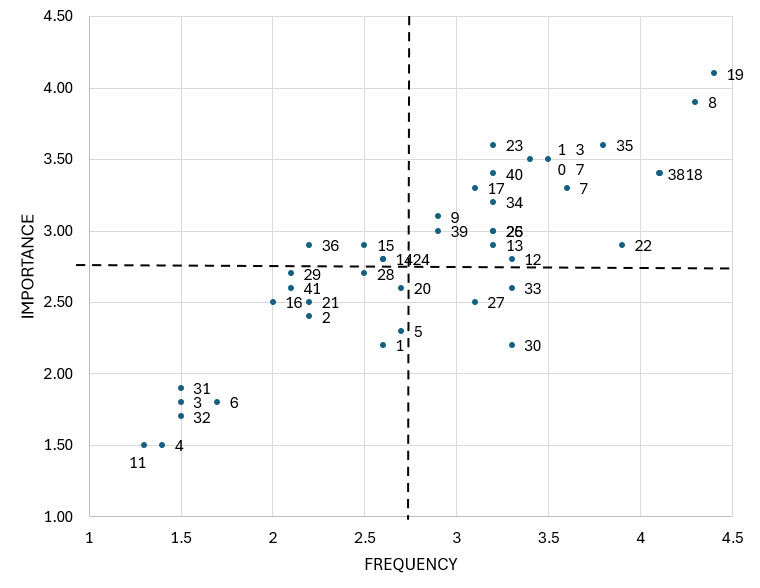
Go Zone of clusters 1 to 8. The dashed lines correspond to the mean value of importance and frequency of all statements.

As shown in Figure 1 and Table 2, statements 8 (“To have a good time”) and 19 (“Have fun”) obtained the highest mean scores for both frequency and importance. Other statements located within the go-zone (Figure 2), indicating above-average ratings on both dimensions, included 18 (“Doing it with friends”), 38 (“To avoid thinking about problems”), 23 (“To have more freedom”), and 10 (“Lose your shame”). In contrast, the lowest mean scores were observed for statement 4 (“Influenced by advertisements”) and statement 11 (“Song lyrics influence”). These were followed by statement 3 (“To follow the example of influencers”), statement 31 (“It’s in the movies”), and statement 32 (“Influence of YouTubers”), as illustrated in the go-zone (Figure 2).

As shown in Table 3, when we conducted a separate analysis for girls and boys, differences were identified compared with the overall findings. Among girls, the statements that were most frequently endorsed were 19 (“Have fun”), 8 (“To have a good time”), and 26 (“Living the experience of being drunk”), whereas the most important statements were 19 (“Have fun”), 22 (“The effect it has”), and 38 (“To avoid thinking about problems”). The statements which received the lowest mean scores for both importance and frequency were 11 (“Song lyrics influence”), 4 (“Influenced by advertisements”) and 32 (“Influence of YouTubers”). Among male participants, the most prevalent statements were 19 (“Have fun”), 17 (“To be more open”), and 38 (“To avoid thinking about problems”), while the most recurrent statements were 8 (“To have a good time”), 19 (“Have fun”), and 18 (“Doing it with friends”). The lowest mean importance scores were observed for items 3 (“To follow the example of influencers”), 4 (“Influenced by advertisements”), and 31 (“It’s in the movies”), whereas the lowest frequency scores corresponded to items 11 (“Song lyrics influence”), 6 (“To quench thirst”), and 26 (“Living the experience of being drunk”). At the cluster level, an increase in mean scores for both importance and frequency in Cluster 1 (“Peer Approval”) was observed among girls, whilst a decrease was observed among boys. In a similar vein, Cluster 2 (“Influence”) demonstrated higher mean scores among the female demographic. In Cluster 5, which was designated “Fun”, girls exhibited a tendency to assign greater importance to the statements, while boys demonstrated a higher frequency of ratings. Finally, in Cluster 8 (“Coping”), a decrease in mean frequency scores was observed among the male subjects.

## 4. Discussion

In our study, adolescents’ motivations for alcohol consumption were identified and prioritized using a concept mapping approach, and each statement was assessed in terms of perceived frequency and importance. Overall, the findings indicate that enjoyment and socially driven reasons were rated as the most salient. Statements related to “having fun,” “having a good time,” and drinking with friends obtained the highest mean scores for both frequency and importance, highlighting the central role of enjoyment and peer interaction. Coping-related motives, such as avoiding thinking about problems, and motives linked to disinhibition or increased openness were also rated relatively highly. These results suggest that alcohol use during adolescence is primarily embedded in social and experiential contexts rather than driven by external media influences [27]. In contrast, statements referring to advertising, song lyrics, influences, or portrayals in movies and social media received the lowest ratings in both frequency and importance. Although adolescents may recognize media figures as influential, they are perceived as less salient than immediate peer relationships and social dynamics. Analyses stratified separately for girls and boys revealed only modest differences. While enjoyment-related motives were highly rated by both girls and boys, girls tended to assign greater importance to peer approval and influence-related factors, whereas boys reported slightly higher frequency ratings for fun-oriented motives. Overall, however, the general pattern of motivation remained largely consistent across boys and girls. Finally, a clear alignment between perceived importance and reported frequency across statements was observed, suggesting that motivations considered more important were also those more frequently endorsed. Together, these findings underscore the predominance of social and hedonic drivers in adolescents’ perceptions of alcohol use.

### 4.1. Social Pressure

The findings of our study serve to reinforce the central role of social dynamics in the consumption of alcohol by adolescents. During this developmental stage, peer groups are the primary source of social reference, and behaviors displayed by peers are frequently internalized and reproduced [11], including patterns of alcohol consumption [19]. In our study, motivations such as “Doing it with friends” or “To make it with Friends” are indicative of this social influence, emphasizing the significance of shared experiences in drinking contexts. This form of imitative behavior can be conceptualized as a strategy for social acceptance and belonging, aimed at avoiding exclusion within peer relationships [13,14,23]. In line with this, the statement ‘fitting into society’ highlights the effect that social integration processes have on the drinking behavior of adolescents.

### 4.2. Fun

Enjoyment-related motives were also prominent. A substantial evidence base identifies pleasure and entertainment as primary drivers of alcohol use among the young [13,19], a pattern that was mirrored in our findings. Statements such as “To have a good time” and “To have fun” suggest that alcohol is perceived as a facilitator of enjoyment and social bonding. This perception may be reinforced by the widespread social normalization of alcohol, particularly in festive, celebratory, and leisure contexts, both within peer groups and family environments [14,15]. Furthermore, the robust association between nightlife environments and alcohol consumption reinforces the notion of drinking as an integral component of leisure activities [23].

### 4.3. Disinhibition

Disinhibition and autonomy-seeking were identified as key themes, as adolescents commonly associate alcohol use with increased independence and freedom [27]. Motivations such as “To have more freedom,” “To be more open,” and “Lose your shame” reflect the perception of alcohol as a means of reducing social inhibitions and facilitating interpersonal interaction [13,28]. This may be in part due to the fact that the search for autonomy may coexist with a diminished perception of risk and a false sense of security induced by alcohol’s disinhibiting effects, reinforcing its appeal as a social facilitator [19,27].

### 4.4. Experimentation

Experimentation represents a further fundamental dimension. Adolescence is characterized by developmental change and active exploration of new experiences [3]. Within this context, alcohol use may be conceptualized as part of a broader process of identity formation and boundary testing [13,23,27]. The “Experimentation” cluster identified in our study, incorporating statements such as “For curiosity”, “Living the experience of being drunk”, and “Not to be responsible for my actions”, encapsulates this tendency towards novelty-seeking and a temporary evasion of responsibility, aligning with the extant literature on adolescent risk-taking, which clearly reflects this orientation toward experimentation and the perceived decrease in responsibility for one’s own behaviors.

### 4.5. Influence

It is becoming increasingly evident that adolescents are utilizing social media as a predominant medium for social interaction. As indicated by the preceding research, there is a demonstrable correlation between exposure to alcohol-related content on these platforms and adolescent drinking behaviors. Of particular note is the impact of influencer-generated content, which has been identified as a significant pathway of influence [17,18]. In our study, motivations such as “To follow the example of influencers,” “Influence of YouTubers,” and “Influenced by advertisements” reflect adolescents’ awareness of these dynamics. Nevertheless, despite acknowledging the potential of social media as an influential factor, the participants allocated relatively low importance and frequency scores to these statements. This finding is noteworthy and may indicate not the absence of influence, but rather a limited awareness of more subtle or normalized marketing effects [29,30]. Alcohol-related promotion on social media frequently operates in an indirect manner and may not be consciously perceived by young people. Marketing strategies, such as interactive content, event promotion, memes, contests, and user-generated posts, become embedded within the socio-cultural identities and peer networks of adolescents. The process of socialization that is observed in this context involves the normalization of alcohol use, which is enhanced by an increase in its social desirability, acceptability, and perceived accessibility. These phenomena result in the integration of alcohol into everyday social activities. [17,31,32,33]. These findings underscore the necessity to enhance adolescents’ critical awareness of the potential influence of digital environments on attitudes and behaviors related to alcohol consumption. It is important to note that the influence of contextual exposure extends beyond the domain of social media. Research has also documented the existence of an association between alcohol advertising and environmental cues in settings where adolescents live and socialize, including the density of alcohol outlets and visible signs of consumption [12,16]. In accordance with this standpoint, the motivational factor “Environment that incites consumption” identified in our study suggests that the interaction between physical and social contexts reinforces the perception of alcohol as both accessible and socially acceptable.

### 4.6. Limitations 

Firstly, the sample size was determined by the requirement for parental informed consent rather than a lack of student interest. Approximately 50% of eligible students ultimately participated, providing a meaningful representation of the target population. In the context of concept mapping, however, the sample size is generally less critical than the diversity and relevance of the participant profile. In this sense, it is important to mention that most participants reported abstinence or low levels of alcohol use, and intensive drinking behaviors were uncommon within our sample. Consequently, the ratings of importance and frequency are likely to reflect primarily the perspectives of non-drinkers or moderate drinkers rather than those engaging in heavier alcohol use. Nevertheless, efforts were made to ensure that all participants were encouraged to contribute during the idea-generation phase, thereby capturing a broad range of perceived motivations regardless of individual drinking behavior. Secondly, the final cluster solution and cluster labels were not validated with the participants. This methodological limitation may have influenced the interpretative validity of the thematic structure. Nevertheless, the cluster nomenclature was derived from the labels originally proposed by adolescents during the grouping phase, thereby ensuring close alignment with their perspective, and the results are aligned with other studies [7]. Notwithstanding the limitations mentioned above, a key strength of our study is the application of concept mapping. This approach combines qualitative exploration with quantitative structuring, thereby producing systematic and visually interpretable representations of complex social phenomena. This approach facilitated the articulation of adolescents’ perspectives in a participatory manner, while concurrently enabling the identification and quantification of perceived motivations. Consequently, the methodology captured a broad spectrum of experiences and provided a structured framework for identifying priority areas for prevention and intervention [26].

## 5. Conclusions

In conclusion, the utilization of concept mapping empowered us to capture adolescents’ perspectives in a nuanced and participatory manner, thus offering insights that extend beyond those typically obtained through survey-based approaches. Despite the necessity for cautious interpretation due to the contextual and relatively small sample size, the findings align with evidence from studies conducted among young adults, suggesting that common social and motivational drivers of alcohol use may operate across developmental stages. These results emphasize the necessity for prevention strategies that explicitly address the social dimensions of alcohol consumption during adolescence. Interventions should prioritize the promotion of accessible, alcohol-free recreational environments, the integration of social skills and emotional regulation training within school settings, and the active involvement of adolescents in the design of preventive initiatives. Policies that foster inclusive leisure opportunities and peer-led engagement may further contribute to reshaping the social context in which adolescent alcohol use occurs.

## Figures and Tables

**Table 1:** Description of the sample [n, (%)].

	Variable	Girls (n = 23)	Boys (n = 15)	*p*
Living together	Parents	6 (26.1)	2 (13.3)	0.598
	Parents and siblings	14 (60.9)	10 (66.7)	
	Others	3 (13.0)	3 (20.0)	
Self-perceived health	Very good	10 (43.5)	8 (53.3)	0.755
	Good	10 (43.5)	6 (40.0)	
	Regular	3 (13.0)	1 (6.7)	
Frequency of drinking alcohol	Never	14 (60.9)	10 (71.4)	0.394
	1 or less per month	3 (13.0)	3 (16.2)	
	2 to 4 glasses per month	4 (17.4)	0 (0.0)	
	2 to 3 drinks per week	2 (8.7)	1 (7.1)	
	4 or more drinks per week	0 (0.0)	0 (0.0)	
Alcohol units per drinking day	None	15 (65.2)	10 (66.7)	0.251
	1 or 2	3 (13.0)	3 (20.0)	
	Between 3 and 4	3 (13.0)	0 (0.0)	
	Between 5 and 6	0 (0.0)	1 (6.7)	
	Between 7 and 9	2 (8.7)	0 (0.0)	
	10 or more	0 (0.0)	1 (6.7)	
Binge drinking	Never	17 (73.9)	13 (86.7)	0.641
	Less than once a month	3 (13.0)	1 (6.7)	
	Monthly	3 (13.0)	1 (6.7)	
	Weekly	0 (0.0)	0 (0.0)	
	On a daily or almost daily basis	0 (0.0)	0 (0.0)	

**Table 2:** Clusters 1 to 8 and their scores according to the criteria of frequency (1 never–5 always) and importance (1 very little–5 very much). Mean: Mean rating; SD: Standard deviation.

ID	Statement	Frequency		Importance	
		Mean	SD	Mean	SD
Cluster 1. Peer Approval					
1	To look older	2.60	1.26	2.20	1.23
2	To think oneself superior	2.20	1.14	2.40	1.58
9	More flirting	2.90	1.10	3.10	1.66
21	Playing the pimp	2.20	1.48	2.50	1.72
24	To draw attention	2.60	1.07	2.80	1.32
29	As a symbol of rebellion	2.10	1.37	2.70	1.57
36	To gain popularity	2.20	1.23	2.90	1.20
Cluster 2. Influence					
3	To follow the example of influencers	1.50	0.71	1.80	1.48
4	Influenced by advertisements	1.40	0.70	1.50	0.97
11	Song lyrics influence	1.30	0.48	1.50	0.85
28	The influence of friends or older siblings	2.50	1.08	2.70	1.34
30	Environment that incites consumption (discotheques, pubs, …)	3.30	1.57	2.20	1.48
31	It’s in the movies	1.50	0.85	1.90	1.20
32	Influence of youtubers	1.50	0.71	1.70	1.34
Cluster 3. Enjoyment					
5	The taste (tastes good)	2.70	0.95	2.30	1.06
6	To quench thirst	1.70	0.67	1.80	0.79
33	I like to use it from time to time	3.30	1.06	2.60	1.17
Cluster 4. Experimentation					
7	For curiosity	3.60	0.52	3.30	1.63
26	Living the experience of being drunk	3.20	1.14	3.00	1.63
39	Not to be responsible for my actions	2.90	0.88	3.00	1.25
Cluster 5. Fun					
8	To have a good time	4.30	0.82	3.90	1.10
19	Have fun	4.40	0.84	4.10	1.29
22	The effect it has	3.90	1.29	2.90	1.29
35	It creates funny moments and situations	3.80	0.79	3.60	1.35
Cluster 6. Disinhibition					
10	Lose your shame	3.40	1.17	3.50	1.08
17	To be more open	3.10	0.88	3.30	1.42
23	To have more freedom	3.20	1.03	3.60	0.97
27	Be more honest	3.10	0.99	2.50	1.27
37	To dare to do	3.50	0.85	3.50	0.71
Cluster 7. Social Pressure					
12	To fit into society	3.30	0.95	2.80	1.40
13	Social pressure	3.20	1.23	2.90	1.45
18	Doing it with friends	4.11	0.78	3.40	1.26
20	It’s something you must do if you go out	2.70	1.42	2.60	1.17
34	To make friends	3.20	1.40	3.20	1.32
41	Competition, not to be left behind if someone else does it	2.10	0.99	2.60	1.35
Cluster 8. Coping					
14	Addiction	2.60	1.17	2.80	1.23
15	Depression	2.50	0.85	2.90	1.29
16	Loneliness	2.00	1.05	2.50	1.35
25	To drown sorrows	3.20	1.14	3.00	1.25
38	To avoid thinking about problems	4.10	0.88	3.40	0.97
40	Being in a bad time in life	3.20	1.03	3.40	1.35

**Table 3:** Clusters 1 to 8 and their scores according to the criteria of frequency (1 never–5 always) and importance (1 very little–5 very much) based on gender. Mean: Mean rating; SD: Standard deviation.

		**GIRLS**				**BOYS**			
**ID**	**Statement**	**Frequency**		**Importance**		**Frequency**		**Importance**	
		**Mean**	**SD**	**Mean**	**SD**	**Mean**	**SD**	**Mean**	**SD**
Cluster 1. Peer Approval									
1	To look older	3.17	1.17	2.50	1.05	1.75	0.96	1.75	1.50
2	To think oneself superior	2.50	1.22	2.67	1.37	1.75	0.96	2.00	2.00
9	More flirting	2.67	0.52	3.33	1.86	3.25	1.71	2.75	1.50
21	Playing the pimp	2.83	1.60	2.83	1.60	1.25	0.50	2.00	2.00
24	To draw attention	3.00	0.89	3.00	1.10	2.00	1.15	2.50	1.73
29	As a symbol of rebellion	2.67	1.51	3.17	1.17	1.25	0.50	2.00	2.00
36	To gain popularity	2.83	1.17	2.83	1.17	1.25	0.50	3.00	1.41
Cluster 2. Influence									
3	To follow the example of influencers	1.83	0.75	1.83	1.60	1.00	0.00	1.75	1.50
4	Influenced by advertisements	1.67	0.82	1.33	0.52	1.00	0.00	1.75	1.50
11	Song lyrics influence	1.33	0.52	1.50	0.84	1.25	0.50	1.50	1.00
28	The influence of friends or older siblings	2.83	1.17	3.17	1.17	2.00	0.82	2.00	1.41
30	Environment that incites consumption (discotheques, pubs, …)	3.67	1.51	2.83	1.60	2.75	1.71	1.75	1.50
31	It’s in the movies	1.83	0.98	2.00	1.10	1.00	0.00	1.75	1.50
32	Influence of youtubers	1.67	0.52	1.50	0.84	1.25	0.50	2.00	2.00
Cluster 3. Enjoyment									
5	The taste (tastes good)	2.83	0.75	2.67	1.21	2.50	1.29	1.75	0.50
6	To quench thirst	1.83	0.75	2.00	0.89	1.50	0.58	1.50	0.58
33	I like to use it from time to time	3.00	0.89	2.67	0.82	3.75	1.26	2.50	1.73
Cluster 4. Experimentation									
7	For curiosity	3.83	0.41	3.83	1.17	3.25	0.50	2.50	0.58
26	Living the experience of being drunk	3.83	0.75	4.00	1.26	2.25	0.96	1.50	0.58
39	Not to be responsible for my actions	2.67	0.52	2.50	0.84	3.25	1.26	3.75	1.50
Cluster 5. Fun									
8	To have a good time	4.00	0.89	4.00	1.26	4.75	0.50	3.75	0.96
19	Have fun	4.17	0.98	4.17	1.33	4.75	0.50	4.00	1.41
22	The effect it has	4.17	1.33	3.50	1.22	3.50	1.29	2.00	0.82
35	It creates funny moments and situations	3.82	0.75	4.00	1.10	3.75	0.96	3.00	1.63
Cluster 6. Disinhibition									
10	Lose your shame	3.67	1.51	3.33	1.21	3.00	1.15	3.75	0.96
17	To be more open	3.00	1.10	2.83	1.33	3.25	0.50	4.00	1.41
23	To have more freedom	3.00	1.10	3.67	0.82	3.50	1.00	3.50	1.29
27	Be more honest	2.83	0.75	2.33	1.51	3.50	1.29	2.75	0.96
37	To dare to do	3.67	1.03	3.33	0.82	3.25	0.50	3.75	0.50
Cluster 7. Social Pressure									
12	To fit into society	3.50	1.05	3.00	1.55	3.00	0.82	2.50	1.29
13	Social pressure	3.33	1.21	3.00	1.67	3.00	1.41	2.75	1.26
18	Doing it with friends	3.60	0.55	3.17	0.75	4.75	0.50	3.75	1.89
20	It’s something you must do if you go out	2.67	1.51	2.50	1.38	2.75	1.50	2.75	0.96
34	To make friends	3.00	1.26	3.00	1.10	3.50	1.73	3.50	1.73
41	Competition, not to be left behind if someone else does it	2.33	1.03	2.67	1.03	1.75	0.96	2.50	1.91
Cluster 8. Coping									
14	Addiction	3.17	0.98	2.67	0.82	1.75	0.96	3.00	1.83
15	Depression	2.83	0.75	3.00	0.63	2.00	0.82	2.75	2.06
16	Loneliness	2.50	1.05	2.50	1.05	1.25	0.50	2.50	1.91
25	To drown sorrows	3.50	0.55	2.67	0.82	2.75	1.71	3.50	1.73
38	To avoid thinking about problems	4.00	0.89	3.00	0.63	4.25	0.96	4.00	1.15
40	Being in a bad time in life	3.33	0.82	3.17	0.98	3.00	1.41	3.75	1.89

## Data Availability

The data are available for consultation upon reasonable request to the corresponding author.
